# Thyroid Hormone Action on Innate Immunity

**DOI:** 10.3389/fendo.2019.00350

**Published:** 2019-06-04

**Authors:** María del Mar Montesinos, Claudia Gabriela Pellizas

**Affiliations:** Facultad de Ciencias Químicas, Centro de Investigaciones en Bioquímica Clínica e Inmunología (CIBICI-CONICET) and Departamento de Bioquímica Clínica, Universidad Nacional de Córdoba, Córdoba, Argentina

**Keywords:** thyroid hormones, innate immunity, neutrophils, natural killer cells, macrophages, dendritic cells

## Abstract

The interplay between thyroid hormone action and the immune system has been established in physiological and pathological settings. However, their connection is complex and still not completely understood. The thyroid hormones (THs), 3,3′,5,5′ tetraiodo-L-thyroxine (T4) and 3,3′,5-triiodo-L-thyronine (T3) play essential roles in both the innate and adaptive immune responses. Despite much research having been carried out on this topic, the available data are sometimes difficult to interpret or even contradictory. Innate immune cells act as the first line of defense, mainly involving granulocytes and natural killer cells. In turn, antigen presenting cells, macrophages and dendritic cells capture, process and present antigens (self and foreign) to naïve T lymphocytes in secondary lymphoid tissues for the development of adaptive immunity. Here, we review the cellular and molecular mechanisms involved in T4 and T3 effects on innate immune cells. An overview of the state-of-the-art of TH transport across the target cell membrane, TH metabolism inside these cells, and the genomic and non-genomic mechanisms involved in the action of THs in the different innate immune cell subsets is included. The present knowledge of TH effects as well as the thyroid status on innate immunity helps to understand the complex adaptive responses achieved with profound implications in immunopathology, which include inflammation, cancer and autoimmunity, at the crossroads of the immune and endocrine systems.

## Introduction

Growing evidence compiled over recent decades has revealed a bidirectional crosstalk between thyroid hormones (THs) and the immune system. This interplay has been demonstrated for several pathophysiological conditions of the thyroid functioning and the innate and adaptive immunity. Many situations primarily affecting the action of THs have an impact on the characteristics and/or functions of immune cells, and are translated to host defense status and related disorders. In turn, immune-related disorders conduct to the most frequent thyroid dysfunctions, which have an autoimmune origin. The connection between these systems is complex and not well-understood. This article reviews the current evidence supporting the contribution of THs to the modulation of innate immunity at the cellular level.

### Thyroid Hormone Action

THs exert a pivotal role for normal development and function. The thyroid produces 3,3′,5,5′ tetraiodo-L-thyroxine (T4) and 3,3′,5-triiodo-L-thyronine (T3), mainly under thyrotropin (TSH) regulation. While this gland secretes 100% of circulating T4, it provides only a low percentage of serum levels of the most physiologically active TH: T3, which for the major part derives from peripheral 5′ deiodination of T4 (1). At the target cell level, the action of THs is genomic (nuclear) and non-genomic. The former requires T3 and the specific nuclear receptors (TRs): TRα1, TRβ1, TRβ2, and TRβ3 (2) and is controlled by a multiprotein complex comprising both corepressors and coactivators (3).

Translocation of TRs from their synthesis in the cytosol to the nucleus is a functionally active process (4). In this regard, non-genomic effects exerted intracellularly by TRs and truncated variants occur rapidly, can be observed in the cytoplasm, mitochondria and other organelles, and are independent of nuclear receptor activity and protein synthesis. Many effects conducted by cytoplasmic TRs involve PI3K-dependent Akt activation (5). Furthermore, non-genomic actions of THs are also initiated at the plasma membrane through different proteins. The best studied is the integrin α*νβ*3, which binds mainly T4 and tetraiodothyroacetic acid (tetrac), a derivative of T4, inducing activation of AMPK, PI3K/Akt, and MAPK (6, 7). Overall, THs interact with a wide variety of signaling pathways that are not yet fully deciphered.

Circulating levels of THs are not representative of what each cell type detects. Instead, the action of THs requires an appropriate interplay among membrane TH transporters, TH deiodinases and TR expression, and thus there is a fine-tuned cellular TH responsiveness. The main TH transporters include monocarboxylate transporters (MCT) 8 and 10, organic anion transporter polypeptides (OATPs) and large neutral amino acid transporters (LATs), with MCT8, MCT10, and LATs having a higher affinity for T3 than T4 uptake. Additionally, the cellular concentrations of THs are regulated by the activity of the 1, 2, and 3 iodothyronine deiodinases: D1, 2, and 3. D2 is an “activating” enzyme, responsible for the peripheral production of 50–80% of the body pool of T3 from T4. In contrast, D3 restrains T3 action, converting T4 and T3 into inactive metabolites. TH transporters and deiodinases exhibit a particular expression profile that is cellular and metabolic state specific (8, 9). Newly discovered actions of T4 and T3 metabolites, such as 3,5-diiodothyronine (3,5-T2), and 3-iodothyronamine (T1AM) are emerging (10).

### Innate Immunity

The immune system includes cells that protect the organism from foreign antigens, such as microbes, cancer cells, toxins, and damage signals. It is simplistically referred to as innate and adaptive immunity. The former offers immediate protection against intruders, with specific cells being able to fight a wide range of pathogens, with the latter being specific and antigen-dependent (11). Moreover, adaptive immunity is orchestrated and directed by its innate counterpart.

The main innate cells are polymorphonuclear leukocytes (PMNL, mainly neutrophils), innate lymphoid cells (ILCs) including natural killer (NK) cells and cytokine-producing helper-like ILCs, innate T-like cells comprising NKT and γδ T cells, monocytes, macrophages and dendritic cells (DCs). Their complete classification and plethora of functions have been extensively reviewed (12–15).

The belief that innate immunity is non-specific was challenged after the description of pattern-recognition receptors and molecules that recognize pathogen and damage-associated molecular patterns from intruders (16, 17). Furthermore, the concept of exclusive memory for adaptive responses was weakened after the description of “trained innate memory,” involving a heightened response upon re-exposure to a certain stimulus (16, 18) under the control of the cellular metabolism (19). Moreover, innate immune tolerance has also been demonstrated (20).

**Figure 1:**
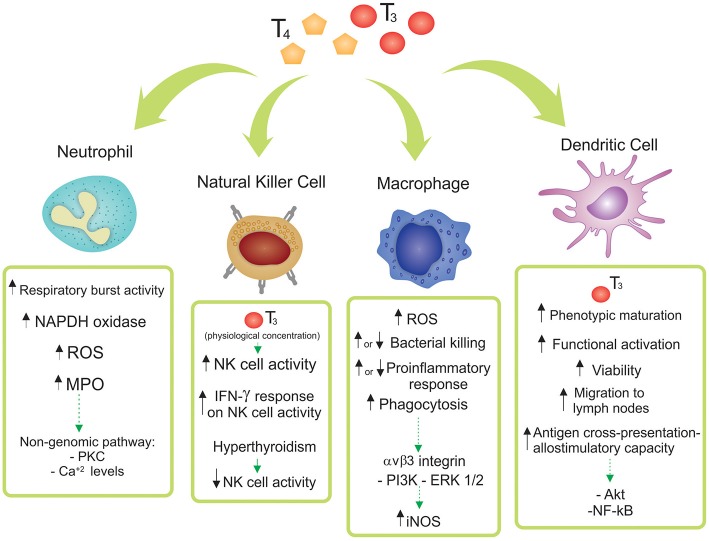
Effects of thyroid hormones 3,3′,5,5′ tetraiodo-L-thyroxine (T4) and 3,3′,5-triiodo-L-thyronine (T3) on innate immune cell subsets. The main reported effects of T3 and/or T4 in Neutrophils, Natural Killer (NK) cells, Macrophages and Dendritic Cells are depicted. Particular differences among the diverse origins of the cells (human, mice, cell lines, and/or tissue source) are shown and discussed in the main text.

This review article focuses on the state-of-the-art of the TH mechanism of action and its effects on innate immunity at cellular level, with the pathophysiological role of the reported findings also discussed. The main effects of T3 and/or T4 in Neutrophils, NK cells, Macrophages and DCs are depicted in Figure 1 and considered below.

### Neutrophils

Neutrophils are the first line of defense against bacteria and fungi, and also help to combat parasites and viruses (21). They travel from the blood to the inflammatory site where they engage and kill microorganisms and clear infections through chemotaxis, phagocytosis, and cytokine synthesis, and the release of reactive oxygen species (ROS) and granular proteins such as myeloperoxidase (MPO) (22). Classical concepts of neutrophil biology are being increasingly challenged by recent findings (23, 24).

Administration of T3 to rats increased the respiratory burst activity of isolated PMNLs with enhanced NADPH oxidase and MPO activities (25, 26). Accordingly, increased mitochondrial oxygen consumption and ROS production were reported in PMNLs from both Graves' disease and toxic adenoma patients (27). Moreover, T3 administration to euthyroid subjects induced ROS generation by PMNLs (28). However, a decrease in oxidative metabolism was registered in human PMNLs during hypothyroidism, which was reversed upon L-T4 substitution therapy (29). The authors suggest that this effect was unlikely to result from direct actions of THs on PMNLs, considering that T3 showed no appreciable effect on superoxide anion (${\text{O}}_{2}^{-}$) generation in *in vitro* experiments with PMNLs from healthy donors. In addition, hypothyroidism causes changes in the lipid composition of PMNLs' membranes that may be involved in their impaired function (30). To note, human neutrophils express TR (31).

T4 and the TH metabolite 3,5-T2 as well as T3 induced respiratory-burst activity and stimulated MPO activity in human PMNLs. These effects were mediated by a non-genomic mechanism initiated at the plasma membrane, dependent on PKC and Ca^+^ levels. Moreover, ${\text{O}}_{2}^{-}$ production in resting PMNLs of hyperthyroid patients was elevated compared with either controls or hypothyroid subjects (32). Furthermore, PMNLs express receptors for T1AM, a T4 derivative, involved in the chemosensory migration toward T1AM (33).

TH metabolism plays an important role in neutrophil function during infection. It has been demonstrated that D3 is strongly expressed in murine neutrophils during chronic chemical inflammation and in acute bacterial infection. Accordingly, human neutrophils express D3, D1, MCT10, and TRα1, which could therefore be involved in TH action in this cell type. Furthermore, evidence has supported the notion that D3 plays a role in the bacterial killing capacity of neutrophils, either through generation of iodide for the MPO system or through modulation of intracellular TH bioavailability (34). Recent results have demonstrated that intracellular TH levels are regulated by D3, playing a key role in neutrophil function in zebrafish, mice and humans (35).

### Natural Killer Cells

NK cells mediate cytolytic activities against tumor and virus-infected targets. Of note, NK cells also possess traits of adaptive immunity and can acquire functional qualities associated with immunological memory (36). The studies of the effects of THs on these cells have produced conflicting results. A positive correlation between serum T3 concentration and NK cell activity in healthy elderly subjects was recorded but exogenous T3 administration increased NK cell activity only in old individuals who had T3 concentrations at the lower end of the reference range (37). Although NK cell functionality was impaired in Graves' patients and restored in the euthyroid state (38, 39), *in vitro* treatment with T4 to peripheral blood lymphocytes from these patients did not show any increase in NK cell activity (40). In agreement, hyperthyroxinemia induced in mice reduced NK cell capacity to lyse target cells (41) whereas exogenous T4 or T3 administered to mice increased NK cell lytic activity (42), as well as during protein starvation (43), or aging (44).

Endogenous IFN-γ plays a relevant role in the host defense against infectious and neoplastic diseases by mechanisms that involve modulation of the NK cell function (45, 46). Both T3 and T4 boosted IFNγ-response in murine NK cells (44, 47), while T4 amplified the effect induced by both IFN-γ and IL-2 (48). These findings suggest a role for THs in the modulation of NK cell sensitivity to IFN-γ.

A recent study linked uterine NK cells (the most prominent leukocytes at the maternal-fetal interface) with THs. These cells express MCT8 and MCT10, as well as TRα1 and β1 in the first trimester of human pregnancy. An increase of IL-6 secretion after T3 exposure *in vitro* was also reported (49).

### Monocytes—Macrophages

Macrophages are strategically positioned in all tissues of the body and can recognize and remove pathogens, toxins, cellular debris, and apoptotic cells. Tissue-resident macrophages in adulthood rely on replenishment by bone marrow (BM)-derived blood monocytes, with circulating monocytes being recruited to tissues by specific chemotactic factors. Among other names, tissue-resident macrophages are referred to as “microglia” in the central nervous system and “Kupffer cells” in the liver (50–52). Depending on the signal and the dose, a second stimulation can result in tolerance or trained immunity (53, 54). In response to stimuli, differentiated macrophages polarize to classically activated M1 or alternatively activated M2 macrophages, although a spectrum of phenotypes across the M1/M2 continuum is recognized. M1 macrophages phagocytize and destroy microbes, eliminate tumor cells, and present antigens to T cells through ROS production, expression of inducible nitric oxide synthase (iNOS) and release of proinflammatory cytokines, thereby promoting T helper (Th) 1 responses (55). In contrast, M2 macrophages show an immunosuppressive phenotype characterized by a decreased antigen presentation to T cells and production of cytokines that stimulate Th2 responses. These regulatory cells are involved in tissue repair, promote tumor growth and exert antiparasitic effects (56).

In spite of controversial results concerning the expression of TR isoforms, macrophages express TRα and β (57–61). In addition, murine and human macrophage cell lines express D2, MCT10, and MCT8 (59). Over the past decade, it has become clear that shifts in cellular metabolism are determinants of macrophage function and phenotype (62). The activities of key enzymes of glycolysis are regulated by THs in these cells, affecting macrophage metabolism and function (63). Stimulation of the immune system in hyperthyroid rats revealed that monocyte migration and ROS production by macrophages were suppressed. In contrast, hypothyroidism enhanced ROS release, whereas monocyte migration was not affected (64).

THs enhanced the phagocytic activity of intraperitoneal macrophages from hypothyroid rats (64). Moreover, T4 administration to old mice also increased their phagocytic capacity (65). In agreement, a stimulatory effect of T4 (but not T3) on the phagocytosis process of cultured peritoneal mouse macrophages was reported (66). However, both THs enhanced bacteria-cell interaction and intracellular killing in mice RAW 264.7 and human THP-1 monocyte-derived macrophage cell lines (67). This mechanism involved the integrin αvβ3, TH-induced iNOS expression, generation of NO and triggering of the PI3K and ERK1/2 signaling pathways.

The inflammatory response exerted by macrophages was stimulated during hypothyroid condition and inhibited in the course of hyperthyroidism (68). T4 inhibited the migration inhibitory factor (MIF) in macrophages (67, 69), and in agreement, low plasma T4 concentrations augmented plasma MIF levels in both patients and rats with severe sepsis (69). Although T4 attenuated proinflammatory responses *in vivo*, no significant changes in IL-6 and TNFα levels could be detected in T4-treated peritoneal macrophages from mice, or in mouse and human cell lines (67).

The “euthyroid sick syndrome” (or “nonthyroidal illness”) is distinctive of critically ill patients with severe infections or sepsis, being characterized by low serum T3 and in serious cases by also low serum T4 without the expected increase in TSH (70). Interestingly, supplementation of T4 to rats and mice in bacterial infectious models enhanced animal survival and attenuated septicemia and inflammatory responses (67, 71). In agreement, hypothyroid mice exhibited increased mortality during inflammation induced by LPS, whereas circulating T3, through TRβ1 signaling, protected animals from endotoxemia (57). However, it was reported that hyperthyroidism increased mice mortality in response to LPS. Noteworthy, Signal Transducer and Activator of Transcription 3 (STAT3) activation induced by LPS or IL-6 was inhibited by T3 through TR signaling in RAW 264.7 cells and in primary cultures of BM-derived macrophages. These authors suggested that inhibition of IL-6 signaling induced by T3 has potent regulatory functions during infection and inflammation (72).

Switching from M1 to the M2 phenotype protects the organism from excessive inflammation, whereas switching from M2 to M1 prevents allergic and asthmatic Th2 reactions, decreases the bactericidal properties of macrophages and favors the resolution of inflammation (63). In this regard, T3 reduced monocyte differentiation into macrophages and induced a M1 signature. In agreement, T3 decreased the expression of genes regulated by M2-activated macrophages through a TRβ1-mediated mechanism (58). Although comparable results were registered in RAW264.7 macrophages, a TRα-dependence was revealed (73). In contrast, in a model of kidney obstruction, ligand-bound TRα inhibited the NF-κB pathway and proinflammatory cytokines in macrophages isolated at the inflammatory site (61).

The role of intracellular TH metabolism in macrophages has been extensively reported and reviewed by Boelen group (34), and is therefore not covered in this review. More recently, a reduction of intracellular T3 concentration due to a lack of D2 activity with impaired macrophage function was reported. Also, primary BM-derived macrophages treated with LPS decreased phagocytosis and proinflammatory cytokines in D2 KO mice (73), consistent with earlier results in RAW264.7 cells (59).

Modifications in the homeostatic conditions of the nervous tissue promote microglia activation, release of inflammatory mediators and phagocytosis of degenerating cells (74). Lima et al. (75) reported that rat microglial cells in culture express TRα and TRβ, whereas other authors did not observe the latter (76). αVβ3 integrin has also been described in these cells (77), and in mice microglia, the TH transporters OATP4A1, LAT2, and MCT10 were also found (78, 79). It is known that T3 modulates microglial development (75) and functions such as migration and phagocytosis by genomic and non-genomic pathways (80). The molecular mechanism involves T3 uptake by TH transporters and binding to TRs, thus triggering multiple signaling pathways (80, 81). Moreover, T3 increased the release of soluble factors by the microglia through STAT3 activation, promoting glioma growth (82).

Liver is one of the most relevant TH target tissues. T3 induced acceleration of cellular ${\text{O}}_{2}^{-}$ consumption, resulting in elevated ROS and NO (83). In agreement, T3-stimulated free radical activity reduced the cellular antioxidant defenses leading to oxidative stress in rats, a phenomenon also observed in human hyperthyroidism (84, 85). Kupffer cells are main scavengers constantly clearing gut-derived pathogens from the blood, preventing liver diseases (86). T3 promoted hyperplasia and hypertrophy of these cells, with a resulting enhancement in the respiratory burst activity. Furthermore, T3-induced calorigenesis resulted in transient elevations in serum TNF-α, determined by actions exerted in Kupffer cells and involving activation of NF-kB (87). The hepatic response induced by T3 involved cell proliferation associated with TNF-α generation by Kupffer cells (88).

### Dendritic Cells

DCs are the main antigen presenting cells in the interface between innate and adaptive immunity. They integrate signals derived from infection or damage, and present processed antigen to naive T cells to tailor the appropriate T cell program. Recent advances in DC immunobiology have led to a clearer understanding of how T cell responses are shaped (89). The main DCs include conventional (classical or myeloid) DCs (cDCs, referred as DCs from now on) and plasmacytoid DCs (pDCs). The genetic signature of DCs from different tissues is similar, but differs from that of pDCs, monocytes and macrophages. To note, DCs are functionally different to macrophages (89, 90). Immature DCs (iDCs) have substantial endocytic activity but lower surface expression of major histocompatibility complex (MHC) class I and II proteins. After encountering any stimulus, DCs mature to undergo considerable cytoplasmic reorganization, transporting peptide-MHC complexes to the cell surface and upregulating costimulatory molecules (90). Recent studies highlighted the relevance of DC migration in the maintenance of immune surveillance. Immature DCs are rather immotile, and after processing foreign and self-antigens or damage signals undergo an activation process, leading to an increase in motility corresponding to upregulation of CC-chemokine receptor 7 (CCR7). The interaction of CCR7 with its ligand guides DCs toward secondary lymphoid organs (91).

**Figure 2:**
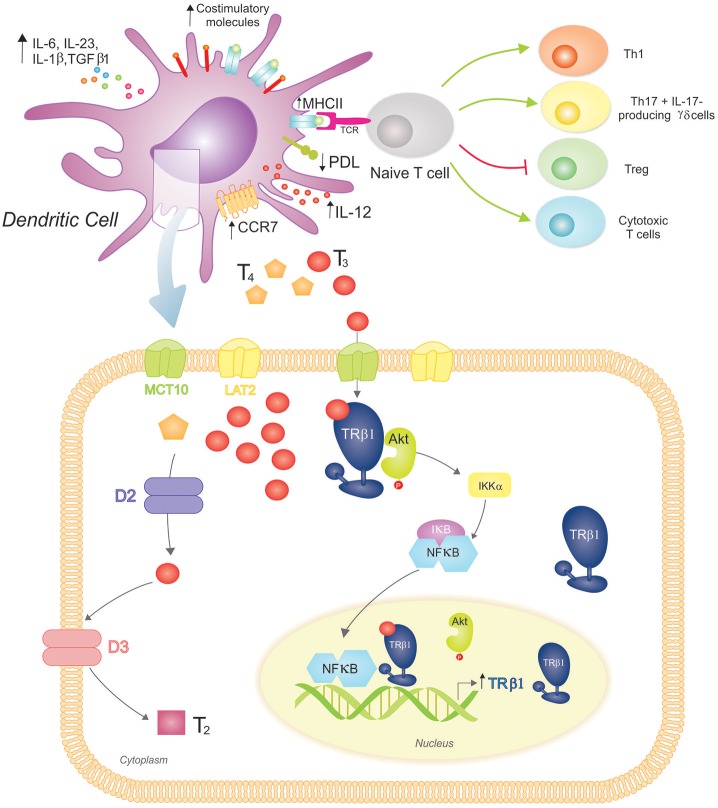
3,3′,5-triiodo-L-thyronine (T3) promotes Dendritic Cell (DC) maturation and function, driving proinflammatory and cytotoxic adaptive responses. **(Top)** T3 promotes DC phenotypic maturation upregulating MHCII and costimulatory molecules. The functional DC activation promotes a proinflammatory cytokine phenotype (increased production of IL-12, IL-6, IL-23, IL-1β, and TGFβ1) that drives adaptive responses favoring the development of Th1 and Th17 T cells, IL-17-producing γδ T cells, and cytotoxic T cells. In contrast, the Treg population is restrained. T3-conditioned DCs also augment CCR7 expression, which favors their migration to lymph nodes, where they present processed antigens in the context of MHCII to specific T cell receptors (TCR) from naïve T cells. T3 also modulates the immune checkpoint, reducing PDL expression on DCs and triggering the down-regulation of PD-1-expressing T cells (not shown). **(Bottom)** DCs take up T3 more effectively than T4 through MCT10 and LAT2. Inside DCs, D2 catalyzes the conversion of T4 to T3, whereas D3 inactivates T3 resulting in T2. These cells mainly express TRβ1 with a preferred cytoplasmic localization, where it co-localizes with Akt. Upon T3 binding to TRβ1, Akt is activated and translocated to the nucleus. This mechanism includes IκB degradation and thus NF-κB cytoplasmic-nuclear shuttling that acts as a transcription factor upregulating TRβ1 expression. An intact T3-TRβ1 signaling is essential for T3-dependent DC induced effects.

The role of THs in the initiation of adaptive immunity remained uncertain for many years, with Mooij et al. providing the earliest clues that THs and other iodinated derivatives, mainly T3, favored the maturation of human peripheral blood monocytes into functional DCs (92). Many years later, our laboratory initiated a study on the effects of THs at the DC level (Figure 2). We observed the expression of TRs in BM-derived mouse DCs, principally the TRβ1 isoform, and mainly in the cytoplasm of both iDCs and LPS-matured DCs. The ability of physiological concentrations of T3 to induce phenotypic and functional activation of DCs and to drive a Th1 profile was also demonstrated (93). Mechanistically, this effect involved activation of the Akt and NF-kB pathways (94) and was counteracted by glucocorticoids (95). The requirement for an intact TRβ-T3 signaling in T3-induced DC activation was confirmed by *in vitro* and *in vivo* studies (94, 96).

Interestingly, we showed that T4, the main circulating TH, did not reproduce T3-dependent effects in DCs. The characterization of the mechanisms of TH transport and metabolism in DCs supports the notion of a homeostatic balance to prevent unspecific systemic activation of DCs. In this regard, DCs express MCT10 and LAT2 TH transporters, and mainly transport T3 with a favored involvement of MCT10, as its inhibition almost prevented T3 saturable uptake mechanism and reduced T3-induced IL-12 production. In addition, DCs express D2 and D3, and exhibit both enzymatic activities with a prevalence toward TH inactivation (97).

Immunotherapy has become the fourth pillar of cancer care, complementing surgery, cytotoxic therapy, and radiotherapy (98). In this context, DCs have been the subject of numerous studies seeking new immunotherapeutic strategies against cancer. However, despite initial enthusiasm, disappointing results including a short half-life of DCs in circulation and induction of tolerogenic responses by death cells, have raised doubts regarding these approaches. Nevertheless, the increased understanding of DC immunobiology and the search for optimization strategies are allowing a more rational development of DC-based immunotherapies (99, 100). A new role for THs in this field has arisen, with T3 binding to TRβ increasing mice DC viability and augmenting CCR7 expression, thereby driving migration of DCs to lymph nodes. Moreover, T3 stimulated the antigen cross-presentation ability of DCs, boosting antigen-specific cytotoxic T-cell responses. Also, vaccination with T3-stimulated DCs in mice bearing B16 melanoma inhibited tumor growth and prolonged host survival (96, 101). Overall, these results established the adjuvant effect of T3-TRβ signaling in DCs, identifying a DC vaccination approach in cancer immunotherapy.

Further recent *in vitro* and *in vivo* evidence has shed light on the molecular and cellular mechanisms driven by T3-conditioned murine DCs (102). Findings revealed an induction of a proinflammatory cytokine profile and a down-modulation of PDL expression in DCs. In co-cultures, these cells increased the frequency of IL-17-producing splenocytes, mainly by the γδ-T population. Thus, down-regulation of tolerogenic T regulatory (Treg) cells and PD1 expression were induced, limiting the inhibitory signals and emphasizing the relevance of T3 as an additional immune-endocrine checkpoint.

The understanding of the effect of THs in human DCs is still limited. Dedecjus et al. (103) reported that the thyrometabolic state influenced the major human peripheral blood DCs, pDCs, and cDCs, with T4 substitution to thyroid cancer patients after surgery increasing the frequency of these cells and the expression of CD86 and HLA-DR (activation markers). In hypothyroid patients with Hashimoto's Thyroiditis, T4 supplementation exerted changes of peripheral blood DC subpopulations, with increased expression of costimulatory molecules (104). Although TRs in human DC populations have not yet been found, increased expression of CD86 by T3 addition to cell cultures of human peripheral blood pDCs was reported (103). Also, T3 increased the ability of human DCs to upregulate the proliferative response and secretion of IL-12 by peripheral blood mononuclear cells, similar to our findings in mice splenocytes co-cultured with T3-stimulated DCs (93).

The proinflammatory role of IL-12 and its involvement in Th1-mediated organ-specific autoimmune diseases (105) confer potential clinical relevance of the aforementioned studies. An increased synthesis of IL-12 by DCs obtained from hyperthyroid mice has been reported (106). Furthermore, patients with Graves' disease exhibited elevated IL-12 circulating levels (107). Considering that DCs are involved in the pathogenesis of autoimmune thyroid diseases (108) and also their potential application for the treatment of these pathologies (109), further research should shed light in this field.

## Concluding Remarks

The relationship between THs and innate immune cells is complex, with an improved knowledge still necessary. Cellular and molecular signaling pathways involved in the crosstalk between THs and innate immune functions, and their role directing adaptive immunity have profound implications in immunopathology, including cancer and autoimmune manifestations of the thyroid gland, at the crossroads of the immune and endocrine systems. The etiopathogenic mechanism involved in both immune-related thyroid pathologies and immune disorders due to thyroid dysfunctions are now better understood. With a focus on particular cell subsets, further research will provide valuable tools for manipulating the immunogenic potential of innate immune cells to positively regulate the development of protective immunity, or negatively control the generation of autoimmune thyroid inflammation.

## Author Contributions

MM and CP: conception and design, analysis and interpretation of available data, writing, review, and revision of the manuscript. MM: design of figures. CP: general supervision.

### Conflict of Interest Statement

The authors declare that the research was conducted in the absence of any commercial or financial relationships that could be construed as a potential conflict of interest.
