# Corrigendum: Thyroid Hormone Action on Innate Immunity

**DOI:** 10.3389/fendo.2019.00486

**Published:** 2019-07-19

**Authors:** María del Mar Montesinos, Claudia Gabriela Pellizas

**Affiliations:** Facultad de Ciencias Químicas, Centro de Investigaciones en Bioquímica Clínica e Inmunología (CIBICI-CONICET) and Departamento de Bioquímica Clínica, Universidad Nacional de Córdoba, Córdoba, Argentina

**Keywords:** thyroid hormones, innate immunity, neutrophils, natural killer cells, macrophages, dendritic cells

An author name was incorrectly spelled as “Claudia Pellizas.” The correct spelling is “Claudia Gabriela Pellizas.”

The authors apologize for this error and state that this does not change the scientific conclusions of the article in any way. The original article has been updated.

